# Health care seeking behavior for diarrhea in children under 5 in rural Niger: results of a cross-sectional survey

**DOI:** 10.1186/1471-2458-11-389

**Published:** 2011-05-25

**Authors:** Anne-Laure Page, Sarah Hustache, Francisco J Luquero, Ali Djibo, Mahamane Laouali Manzo, Rebecca F Grais

**Affiliations:** 1 Epicentre, Paris, France; 2 Ministère de la Santé, Niamey, Niger; 3 Direction Régionale de la Santé Publique, Maradi, Niger

## Abstract

**Background:**

Diarrhea remains the second leading cause of death in children under 5 years of age in sub-Saharan Africa. Health care seeking behavior for diarrhea varies by context and has important implications for developing appropriate care strategies and estimating burden of disease. The objective of this study was to determine the proportion of children under five with diarrhea who consulted at a health structure in order to identify the appropriate health care levels to set up surveillance of severe diarrheal diseases.

**Methods:**

A cluster survey was done on 35 clusters of 21 children under 5 years of age in each of four districts of the Maradi Region, Niger. Caretakers were asked about diarrhea of the child during the recall period and their health seeking behavior in case of diarrhea. A weighted cluster analysis was conducted to determine the prevalence of diarrhea, as well as the proportion of consultations and types of health structures consulted.

**Results:**

In total, the period prevalence of diarrhea and severe diarrhea between April 24^th ^and May 21^st ^2009 were 36.8% (95% CI: 33.7 - 40.0) and 3.4% (95% CI: 2.2-4.6), respectively. Of those reporting an episode of diarrhea during the recall period, 70.4% (95% CI: 66.6-74.1) reported seeking care at a health structure. The main health structures visited were health centers, followed by health posts both for simple or severe diarrhea. Less than 10% of the children were brought to the hospital. The proportion of consultations was not associated with the level of education of the caretaker, but increased with the number of children in the household.

**Conclusions:**

The proportion of consultations for diarrhea cases in children under 5 years old was higher than those reported in previous surveys in Niger and elsewhere. Free health care for under 5 years old might have participated in this improvement. In this type of decentralized health systems, the WHO recommended hospital-based surveillance of severe diarrheal diseases would capture only a fraction of severe diarrhea. Lower levels of health structures should be considered to obtain informative data to ensure appropriate care and burden estimates.

## Background

Although better sanitation, hygiene and access to care have successfully alleviated the burden of diarrheal diseases in developed countries [1,2], diarrhea remains the second leading cause of death in children under 5 years of age in the world, representing nearly one in five child deaths - about 1.5 million each year [3,4]. In sub-Saharan Africa, the etiology of diarrhea is seldom known due to the lack of infrastructure for diagnosis. Further, improved hygiene has not had a major impact on some causes of diarrheal diseases, such as rotavirus, for which vaccination is now the recommended prevention strategy [5-7]. As rotavirus vaccines have become available, considerable efforts have been made to document the burden of severe rotavirus diarrhea with recent focus on Asia and Africa through the Asian and African Rotavirus Networks [8-11]. Despite these efforts, knowledge about the disease burden as well as the circulating strains is still lacking in many countries, such as Niger [8,12,13]. The World Health Organization (WHO) developed a generic protocol for the hospital-based surveillance of severe diarrhea due to rotavirus in children under 5 years of age to inform decision making prior to vaccine introduction [14]. The recommended first step of this protocol is a survey among caretakers of children under 5 years of age to establish their hospital-use pattern in case of diarrhea. A first field-test of the survey protocol in Ghana showed that a hospital-based surveillance of rotavirus would miss the vast majority of children with severe diarrhea [15], emphasizing the need for investigating local specificities before implementing a surveillance system.

The organization of the health system in Niger is based on several international initiatives launched in 1995-1996 [16]. This period was marked by the creation of health districts for decentralization of care, the gradual introduction of the Integrated Management of Childhood Illness (IMCI) algorithms [17] in all districts, and the implementation of cost recovery following the Bamako Initiative [18]. Despite these efforts, the most recent national Demographic and Health Survey (DHS) in Niger in 2006 showed that only 17% of caretakers of children under 5 years of age had sought advice or treatment for childhood diarrhea [19]. With the aim of reducing child mortality in line with the UN Millennium Development Goals, free care for children under 5 years of age was introduced in April 2007. The health system in Niger is pyramidal based on health structures with increasing levels of service: health posts (cases de santé), health centers (centres de santé intégrés), district and regional hospitals [20]. Health posts provide basic care and preventive services and are staffed mainly by community health workers aided by community representatives. Health centers are staffed by nurses and ensure the provision of all services not requiring hospitalization. Diarrhea with severe dehydration is the only severe condition that does not automatically lead to referral to a hospital. In the absence of other severity signs, diarrhea with severe dehydration is managed by injection of intravenous fluids at the health center level.

The objective of this study was to determine the proportion of children under 5 years of age suffering from severe diarrhea, who were treated in the health care system of Maradi region, Niger. This was an initial step to determine the suitable sites to implement a rotavirus and diarrheal disease surveillance system in order to provide essential information for guidance on vaccine introduction.

## Methods

### Study Site

The region of Maradi is comprised of seven health districts with a total estimated population of about 3 million inhabitants in 2009 (Recensement general de la Population, 2001, Institut National de la Statistique). The survey took place in four health districts of the region: the city of Maradi, and rural districts of Madarounfa, Aguié and Guidan Roumdji, with a total population of approximately 1.4 million. The city of Maradi is the third largest in Niger and its economic capital. The public health system coverage reported by the Ministry of Health - i.e. the proportion of the population with access to a health structure, including health post, within a distance of less than 5 km - is 100% in the city of Maradi. Aguié, Madarounfa and Guidan Roumdji are large rural districts covering areas of 2800, 4700, 3500 km^2^, respectively. In 2009, the average number of public health structures in 100 km^2 ^was 1.5 (Madarounfa) to 2.1 (Aguié and Guidan Roumdji) and the public health system coverage was 85%, 83% and 94% in Aguié, Madarounfa and Guidan Roumdji, respectively.

### Study population

All children aged 0 to 59 months resident in the survey districts at the time of the study were eligible for inclusion. The sample was obtained using stratified cluster sampling with each of the three above-mentioned districts and the city of Maradi considered as separate strata. For the sample size calculation, we assumed an alpha error of 0.05, precision of 5%, a design effect of 2 and expected prevalence of severe diarrhea of 30%. Considering these assumptions and including 10% of missing or incomplete data, the minimum sample size was 708 children in each stratum. To obtain the desired sample size we used 35 clusters of 21 children in each stratum.

Clusters were allocated within the four strata proportionally to the population of each village, based on the most recent population census in 2001 adjusted by an annual growth rate (Recensement general de la Population, 2001, Institut National de la Statistique). In the city of Maradi, spatial sampling using a super-imposed grid was used to select the starting point. In the rural districts, we followed the WHO EPI methodology [21].

Households were defined as individuals sleeping regularly under the same roof and sharing meals. As polygamy is common in this region, a wife and her children were each considered a unique household. If more than one household was present in a home, one household was chosen randomly with the aid of a random number table. Similarly, if more than one child aged 0 to 59 months was eligible for inclusion in the selected household; one child was selected at random.

### Data collection

All information was elicited by interviews, which were conducted with the mother or caretaker. A recall period of 27 days (April 24^th ^until May 21^st^) was used. The standardized questionnaire was based on the questionnaire in Annex of the WHO generic protocol for a community-based survey on utilization of health care services for gastroenteritis in children [14]. It was adapted locally after field testing and the following questions were removed for the purpose of simplification: socio-economical status, duration of the disease and some symptoms during illness.

As our primary interest was in seeking care at formal health care structures, respondents reporting that they sought care or intended to seek care at a fixed or mobile pharmacy or drug merchant, or those intending to seek care from family, friends and traditional healers were considered as not intending to seek consultation. These results are thus presented as a reason for not consulting a health care structure.

### Definitions

Following the WHO protocol, we defined acute watery diarrhea as more than 3 watery or liquid stools over a 24-hour period without the presence of blood. Severe watery diarrhea was defined as acute watery diarrhea with the presence of at least two of the following signs: loss of consciousness and/or sunken eyes and/or incapable of drinking or drinking very little. Diarrhea was also considered severe if the child received intravenous rehydration treatment.

### Data entry and analysis

An independent data entry team in Maradi, Niger, entered data using EpiData 3.1 (EpiData Association, Odense, Denmark). Coherency and consistency checks were performed on double entry. Data analysis was done using Stata 10.0 (College Station, TX, USA).

Crude estimates and design effects were obtained considering the survey design [22]. Sampling weights were calculated at each level to account for different household compositions (more than one child eligible for inclusion), as well as for the population of each stratum when compiling results.

### Ethical considerations

This study adhered to the principles that govern biomedical research involving human subjects [23]. Ethical approval was granted by the Ethical Committee of Niger and authorization granted by the Ministry of Health, as well as from the head of each village participating in the survey. Written informed consent was obtained from participants. Children presenting with diarrhea at the moment of the survey or other pathology visibly requiring treatment were referred to the closest health structure.

## Results

The survey took place from 21^st ^to 28^th ^of May 2009. A total of 3134 households were visited in all strata. Of these, 133 (4.2%) households were absent and 61 (1.9%) refused to participate in the survey. In total, 2940 households were included in the survey with 735 included in each stratum. The mean number of children aged 0 to 59 months per household was 2.4 with a male/female ratio of children included in the survey of 1.01. The interview respondent was the mother in 94.4% of households and without prior education in 86.7% of respondents. Demographic characteristics of households included in the survey are presented in Table 1.

**Table 1:** Demographic characteristics of households included in the survey.

		Aguie	Guidan Roumdji	Maradi CU	Madarounfa	Total
Total population						

Number of households/respondents		735	735	735	735	2940
Number of children under 5 yo		1550	2011	1736	1644	6941
Number (%) of households with						
	1 to 2 children < 5 yo	535 (72.8)	463 (63.0)	492 (66.9)	518 (70.5)	2008 (68.3)
	3 to 5 children < 5 yo	192 (26.1)	211 (28.7)	209 (28.5)	200 (27.3)	812 (27.7)
	more than 5 children < 5 yo	8 (1.1)	61 (8.3)	34 (4.6)	17 (2.3)	120 (4.1)
Number (%) of respondents with						
	0 years of education	680 (92.5)	669 (91.0)	534 (72.7)	665 (90.5)	2548 (86.7)
	1 to 6 years of education	35 (4.8)	38 (5.2)	72 (9.8)	39 (5.3)	184 (6.3)
	more than 6 years of education	20 (2.7)	28 (3.8)	129 (17.6)	31 (4/2)	208 (7.1)

Selected population						

Selected children		735	735	735	735	2940
Sex ratio M/F		0.96	0.94	1.03	1.15	1.01
Age						
	median (month) (IQR)	23 (12-35)	23 (13-35)	23 (12-36)	25 (14-38)	24 (13-36)
	< 1 year (%)	214 (29.1)	175 (23.9)	184 (25.2)	153 (20.9)	726 (24.8)
	1 - 2 years (%)	202 (27.5)	212 (29.0)	203 (27.8)	201 (27.5)	818 (28.0)
	2 - 3 years (%)	163 (22.2)	185 (25.3)	169 (23.2)	186 (25.4)	703 (24.0)
	3 - 4 years (%)	106 (14.4)	110 (15.1)	104 (14.2)	117 (16.0)	437 (14.9)
	4 - 5 years (%)	49 (6.7)	49 (6.7)	70 (9.6)	75 (10.2)	243 (8.3)

Maradi Region, Niger, May 2009

### Diarrheal episodes

Of the 2940 children selected for inclusion, 1099 caregivers reported at least one episode of diarrhea during the recall period of 27 days. Of these, 33 were excluded: 4 children did not meet the definition of diarrhea (more than 3 stools per 24 h) and 29 reported blood in the stool. Therefore, the following analyses were done for 1066 reported cases. For the entire survey region, the period prevalence of watery diarrhea was 36.8% (95%CI: 33.7 - 40.0). Prevalence varied among strata (Table 2). More than half of the children aged 6 to 18 months had a diarrheal episode reported during the recall period (Table 2).

**Table 2:** Prevalence of diarrhea cases and severe cases by district, by age and overall in children under 5 years of age.

	All diarrhea cases				Severe cases		
	n	%*	95% CI	deff	n	%*	95% CI
Total (N = 2940)	1066	36.8	[33.7-40.0]	3.2	98	3.4	[2.2-4.6]
By district							
Aguie (N = 735)	250	34.0	[28.2-39.8]	2.9	15	1.5	[0.6-2.5]
Guidan Roumdji (N = 735)	273	40.1	[33.1-47.1]	5.2	28	5.3	[2.2-8.5]
Maradi CU (N = 735)	219	32.0	[27.4-36.7]	2.0	14	1.8	[0.7-2.9]
Madarounfa (N = 735)	324	44.2	[38.7-49.6]	1.2	41	5.5	[3.2-7.8]
By age (months)							
0-2 (N = 100)	19	16.6	[8.1-25.0]	1.3	1	1.5	[0.0-4.4]
3-5 (N = 160)	49	35.0	[22.8-47.1]	3.0	4	3.8	[0.0-9.1]
6-11 (N = 382)	202	54.2	[48.0-60.5]	1.7	17	4.0	[0.2-6.1]
12-17 (N = 206)	203	52.1	[45.8-58.3]	1.7	18	4.0	[0.9-7.0]
18-23 (N = 153)	150	37.3	[30.3-44.3]	2.0	9	1.3	[0.3-2.3]
24-59 (N = 1492)	443	28.8	[25.2-32.3]	2.3	49	3.7	[2.2-5.1]

Maradi Region, April 24^th^- May 21^st ^2009

* Weighted proportions (see methods)

Among the reported symptoms, sunken eyes were mentioned by 74.7% (n = 777/1066), unable to drink or drinking very little by 13.2% (n = 167/1066) of caregivers and loss of consciousness in 5.1% (n = 54/1066). Of the 1066 cases of acute watery diarrhea during the recall period, 9.2% (n = 98/1066) of the children with diarrhea met the criteria for severe watery diarrhea, leading to a period prevalence of 3.4% (95%CI: 2.2-4.6) (Table 2).

### Health care seeking behavior

**Figure 1:**
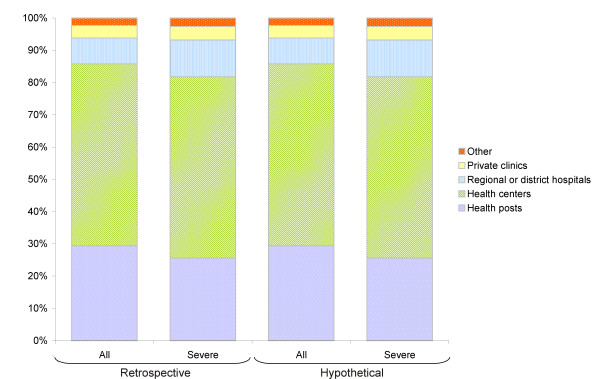
**Distribution of consultations by type of health structure for diarrhea and severe diarrhea cases in children under 5 years of age**. Maradi Region, April 24^th ^- May 21^st ^2009. The bars show the proportion of consultations by type of health structure among all diarrhea cases and severe cases who consulted during the recall period (Retrospective use) and hypothetical consultation location in case of diarrhea or severe diarrhea for children who did not have diarrhea during the recall period (Hypothetical use).

Of those reporting diarrhea during the recall period, 70.4% (95%CI: 66.6-74.1) reported seeking care at a health structure, and among severe cases, 83.8% (95%CI: 75.2-92.4) sought care. Proportions of consultations were consistently high in all districts, ranging from 68.7% (95%CI: 59.1-76.5) in the city of Maradi to 76.4% (95%CI: 70.4-82.5) in Madarounfa for all diarrhea cases and from 66.7% (95%CI: 36.9-96.5) in Aguie to 90.3% (95%CI: 70.8-100) in the city of Maradi for severe cases. In all sites, more than half of all diarrhea cases sought care at health centers and around one-third at health posts (Figure 1).

There was no association between consultations at a health care structure and the level of education of the caretaker (Table 3). Consultations were associated with increasing number of children under 5 years of age in the household. Age was also associated to consultations at a health care structure, with a higher proportion of consultations for children aged 6 to 18 months.

**Table 3:** Proportions of consultations in a health care structure for diarrhea according to demographic characteristics.

	Proportion	Proportion ratio	95% CI	p
Education				
0 years	68.3	Ref		
1 to 6 years	76.4	1.12	[0.98-1.27]	0.098
more than 6 years	63.4	0.93	[0.79-1.09]	0.359
Number of children				
1 to 2	64.5	Ref		
3 to 5	74.9	1.16	[1.07-0.27]	0.001
more than 5	86.0	1.33	[1.18-1.50]	< 0.001
Sex				
male	66.3	Ref		
female	70.8	1.07	[0.98-1.17]	0.151
Age (months)				
24-59	60.9	Ref		
18-23	70.0	1.15	[1.02-1.30]	0.025
12-17	77.8	1.27	[1.15-1.41]	< 0.001
6-11	75.7	1.24	[1.11-1.39]	< 0.001
3-5	73.5	1.21	[1.02-1.42]	0.031
0-2	47.4	0.78	[0.48-1.25]	0.294
Vaccination				
No	67.0	Ref		
Oral history	66.3	0.99	[0.89-1.10]	0.86
Card confirmed	73.1	1.09	[0.99-1.21]	0.091

Maradi Region, April 24^th^-May 21^st ^2009

The main reasons for non-consultation were spontaneous recovery and self-medication, mostly sought from roadside vendors (Table 4). Financial problems were cited in approximately 10% of respondents.

**Table 4:** Reasons for not consulting in a health structure for diarrhea cases in children under 5 years of age (N = 335).

	n	%**	95% CI
Spontaneous recovery	120	43.4	[34.2-52.6]
Pharmacy	64	15.8	[10.7-22.7]
Financial problems	34	13.1	[4.6-21.6]
Self-medication	33	6.6	[4.1-10.6]
Distance	19	6.2	[3.0-12.1]
Inability*	13	5.6	[1.6-9.7]
"Teething" or "newborn" diarrhea	12	3.1	[1.2-5.0]
Traditional healer	10	1.8	[0.4-3.2]
Traditional medicine	11	1.2	[0.3-2.1]

Maradi Region, April 24^th ^- May 21^st ^2009

* Children at home, pregnant, too old

** Weighted proportions (see methods)

### Treatment and Hospitalization

Among all patients who sought medical consultation, 80.4% (95%CI: 76.8-84.1) received oral rehydration solution and 6.1% (95%CI: 3.1-8.7) received intravenous rehydration. Over half of the severe cases (53.3%; 95%CI: 43.2-63.5) received intravenous rehydration.

In total, 50 children with diarrhea were hospitalized (or under observation) for at least one night, representing 8.2% of all diarrhea cases (95%CI: 5.3-11.1). The median duration of hospitalization was 2 nights (IQR: 1-5). Of these, 25 met the definition of severe diarrhea, leading to a proportion of 44.0% (95%CI: 28.8-59.2) of severe diarrhea cases who were hospitalized.

### Hypothetical health seeking behavior

All respondents whose child did not have diarrhea during the recall period were asked what they would do in case their child had diarrhea. Almost all respondents (98.1%; n = 1806/1841) stated that they would seek medical attention for a future episode of diarrhea whether simple or severe. The health structures where people would seek care were the health centers, followed by health posts (Figure 1). The answers were very similar whether the putative diarrhea was simple or severe.

## Discussion

This survey in four health districts in the region of Maradi, Niger, confirms the high burden of diarrhea in Niger while showing an adequate use of the decentralized health system, and provides essential information for the establishment of a surveillance system for diarrheal diseases.

The 36.8% period prevalence of acute watery diarrhea found here is similar to the results of the 2006 DHS survey, when adjusted by the duration of the recall period [19]. In contrast, the percentage of reported health system consultations in case of diarrhea was remarkably higher; 70% overall compared to 17.2% reported consultations at the national level and only 13.7% in the region of Maradi in the 2006 DHS survey. In addition, the proportions were consistently high throughout districts, with no significant differences between the urban district (Maradi CU) and the three rural districts, which is remarkable in a region where reaching a health structure can take up to several hours of walking due to the long distances and lack of public transportation. The interpretation of the increase since 2006 is not straightforward due to potential methodological differences between this and the DHS surveys, in particular in the length of the questionnaire and the recall period. It is interesting to note that the data parallel an approximately 3.5 × increase in the number of consultations for diarrhea in children under 5 years of age reported from the health centers in Niger between 2006 and 2009 [24]. Abolition of user fees for children under 5 years old in April 2007 may be one of the major reasons for this increase. User fees have been shown to have an impact on patient behavior, and in particular on the delay before consultation and in the most vulnerable groups [25,26].

The proportion of patients having received oral rehydration solution shows good compliance with national and international recommendations for the treatment of simple diarrhea [4,27]. However, only half of the cases retrospectively classified as severe received intravenous rehydration therapy. Interestingly, the type of health structure visited did not vary much according to severity, both in the retrospective and hypothetical questionnaire, with health centers as the most frequently visited structures, followed by health posts. Referrals were not precisely documented here, but hospitalizations for at least one night were recorded and reported in only 44% of severe cases. As this also includes patients who stayed overnight at a health center, it is an over-estimate of the proportion of severe cases who were admitted to hospital. This, together with the decentralized healthcare organization and recommendations for treatment of severe dehydration at the health centre level, suggests that the majority of children with severe diarrhea would be missed by a hospital-based surveillance system.

Data from other African settings also show the limits of hospital-based surveillance. Similar low coverage of hospital-based surveillance was also concluded from a survey in Ghana [15]. In a hospital-based study in Kenya, the reported incidence of rotavirus-associated diarrhea was at least 70% higher in the areas close to the hospital than in the district overall [28]. Relying only on hospital based surveillance may have an impact on the estimated incidence of rotavirus-associated diarrhea, but also on the characterization of circulating genotypes, as well as on the estimation of expected health and economic impacts of vaccination, which are essential indicators to guide vaccine introduction [11,29,30].

The principal limitations of this study are related to its retrospective design. The survey was meant to focus primarily on severe diarrhea but retrospective classification of the severity of the disease was made difficult by the subjective assessment of dehydration signs, as shown even among clinicians [31]. Recall bias for diarrhea has been shown to be high after 7 days, with under-reporting up to 45% after this time [32,33]. Positive behavior (here consultation at a health structure) could have been over-reported, although it was mentioned by surveyors that many caregivers showed the health passport of the child to prove the visit at the health structure. Finally, the questionnaire focused on health-seeking behavior of the care provider and was not optimal for documenting subsequent referrals within the healthcare system. Some of these limitations could be addressed in the future by slightly modifying the survey methodology. First and most importantly, severity scales such as the Clark or Vesikari scores might be more appropriate to evaluate severity than dehydration signs. These scores are widely used, in particular as the main outcome to assess rotavirus vaccine efficacy [34]. A modified Vesikari score was proposed and validated for use in outpatient settings, which excludes dehydration signs, considered subjective, in favor of objective signs such as number of stools or vomiting or duration of illness [35]. We suggest that this score be adapted and used for retrospective assessment of diarrhea in the future. Other suggested modifications include recording travel time from the house to nearest health care structure, clarifying questions on different steps in health care seeking behavior (first and subsequent visits, referrals), and, when possible, asking for the health passport to confirm the visit.

## Conclusions

The results of this survey show an increase in health care seeking behavior in case of diarrhea of children under 5 years of age in the Maradi region since the 2006 DHS survey, suggesting the efficacy of recent health policies for children in Niger. In addition, the data suggest that hospital-based surveillance of severe diarrheal diseases might not be appropriate in this type of decentralized health system. Health centers and posts are the cornerstone of the health system, in particular in rural areas, and should not be overlooked for the establishment of surveillance systems.

## Competing interests

The authors declare that they have no competing interests.

## Authors' contributions

ALP had full access to the data, carried out the data analysis and drafted the manuscript. SH participated in the design of the study, carried out the survey and participated in the data analysis. FJL participated in the design of the study, data analysis and interpretation and review of the paper. AD and MLM participated in the interpretation of the data. RFG participated in the study design, data analysis and interpretation, drafting and review of the manuscript, and obtained funding. All authors read and approved the final manuscript.

## Pre-publication history

The pre-publication history for this paper can be accessed here:

http://www.biomedcentral.com/1471-2458/11/389/prepub
